# Higher Circulating miR-199a-5p Indicates Poor Aerobic Exercise Capacity and Associates With Cardiovascular Dysfunction During Chronic Exposure to High Altitude

**DOI:** 10.3389/fphys.2021.587241

**Published:** 2021-02-09

**Authors:** He Huang, Shenwei Xie, Xiaolan Gu, Bin Xiang, Zhifeng Zhong, Pei Huang, Yuqi Gao, Peng Li

**Affiliations:** ^1^Department of High Altitude Operational Medicine, College of High Altitude Military Medicine, Army Medical University (Third Military Medical University), Chongqing, China; ^2^College of High Altitude Military Medicine, Institute of Medicine and Equipment for High Altitude Region, Army Medical University (Third Military Medical University), Chongqing, China; ^3^Key Laboratory of Extreme Environmental Medicine, Ministry of Education of China, Chongqing, China; ^4^Key Laboratory of High Altitude Medicine, PLA, Chongqing, China; ^5^Shigatse Branch, Second Affiliated Hospital (Xinqiao Hospital) of Army Medical University (Third Military Medical University), Tibet, China; ^6^Department of Infectious Diseases, First Affiliated Hospital of Army Medical University (Third Military Medical University), Chongqing, China

**Keywords:** circulating microRNA, miR-199a-5p, biomarker, cardiovascular dysfunction, exercise capacity, hypoxia, high altitude

## Abstract

**Background:**

Hypoxia-induced decline in exercise capacity is ubiquitous among lowlanders who immigrated to high altitudes, which severely reduces their work efficiency and quality of life. Although studies have revealed that hypoxia-induced cardiovascular dysfunction limits exercise capacity at high altitudes, the mechanisms have not been well explored at the molecular level. miR-199a-5p is hypoxia-sensitive and serves as an important regulator in cardiovascular pathophysiology. However, whether miR-199a-5p is involved in cardiovascular dysfunction at high altitudes and contributes to subsequent reductions in exercise capacity remains unknown. Thus, this study aimed at exploring these relationships in a high altitude population.

**Methods:**

A total of 175 lowlanders who had immigrated to an altitude of 3,800 m 2 years previously participated in the present study. The level of plasma miR-199a-5p and the concentration of serum myocardial enzymes were detected by qRT-PCR and ELISA, respectively. Indices of cardiovascular function were examined by echocardiography. The exercise capacity was evaluated by Cooper’s 12-min run test and the Harvard Step Test. Furthermore, we explored the biological functions of miR-199a-5p with silico analysis and a biochemical test.

**Results:**

The level of miR-199a-5p was significantly higher in individuals with poor exercise capacity at 3,800 m, compared with those with good exercise capacity (*p* < 0.001). miR-199a-5p accurately identified individuals with poor exercise capacity (AUC = 0.752, *p* < 0.001). The level of miR-199a-5p was positively correlated with cardiovascular dysfunction indices (all, *p* < 0.001). Furthermore, miR-199a-5p was involved in the oxidative stress process.

**Conclusion:**

In this study, we reported for the first time that the level of circulating miR-199a-5p was positively associated with exercise capacity during chronic hypoxia at high altitudes. Moreover, higher miR-199a-5p was involved in hypoxia-induced cardiovascular dysfunctions, thus contributing to poorer exercise endurance at high altitudes.

## Introduction

With the rapid development of the economy, millions of lowlanders (such as the Chinese Han) have immigrated to high altitude regions (elevation: ≥2,500 m) for work, construction, and military operations. During exposure to high altitude regions, hypoxia-induced decline in exercise capacity is ubiquitous among them, which severely reduces their work efficiency and quality of life (Kayser, 2013; Chatterjee et al., 2017). Subsequently, with the progress of high altitude exposure, some lowlanders could acclimatize well to (or termed as gradually tolerate) high altitude, thus restoring their exercise capacity (Burtscher et al., 2015, 2018; Chapman et al., 2016). The gradual adaptation to high altitude hypoxia is a physiological process encompassing a lot of changes in different body systems to enhance oxygen intake and exchange, the delivery ability of the oxygen transfer system, and the oxygen utilization efficiency of tissues, thus meeting the oxygen demands of the human body and supporting its exercise performance (Lundby and Calbet, 2016). With respect to the exercise capacity, the compensatory regulation of the cardiovascular system is most important due to its central role in the oxygen transfer (Naeije, 2010, 2018; Faoro et al., 2014; Whayne, 2014; Stembridge et al., 2016). Consistently, numerous previous studies have proposed that cardiovascular un-acclimatization leads to lower function of the right ventricular, higher pulmonary artery pressure, and more severe injury of the myocardium, as well as limits the aerobic exercise capacity of humans during chronic exposure to high altitudes (Ortega et al., 2006; Penaloza and Arias-Stella, 2007; Naeije et al., 2010; Stuber et al., 2010; Allemann et al., 2012; Yang et al., 2015; Taylor et al., 2016; Enzhi Feng et al., 2017; Stembridge et al., 2019). However, the mechanisms involved in cardiovascular dysfunction and decreased exercise capacity at high altitudes have not been well explored at the molecular level.

MicroRNAs (miRNAs), which are small non-coding RNA molecules, regulate gene expression post-transcriptionally *via* degrading or blocking mRNA translation. Thus, they play an important role in various physiological and pathophysiological processes, including cardiovascular functions (Hung et al., 2018; Gevaert et al., 2019). Using an assay profiling experiment, we found that several circulating microRNAs evidently changed (foldchange > 2, *p* < 0.01) after entering high altitude (Liu B. et al., 2016; Liu Y. et al., 2016), which indicated that these hypoxia-derived microRNAs might participate in the physiological compensation process at high altitude. According to the list of different microRNAs between patients with heart failure and normal cardiac function (De Rosa et al., 2018), only miR-199a-5p was involved both in heart-failure-related microRNAs and hypoxia-related microRNAs detected by us. The hypoxia-related microRNAs were also analyzed one by one in detail by searching the literature, and miR-199a-5p was found to be most closely related to cardiac function. miR-199a-5p is abundantly expressed in the myocardial tissue, and its sequence is highly conserved among vertebrates (Haghikia et al., 2011; Han et al., 2011). Emerging evidence has proposed that the dysregulation of miR-199a-5p contributes to multiple cardiovascular diseases, such as primary hypertension, pulmonary artery hypertension, gestational hypertension, cyanotic congenital heart disease, cardiac hypertrophy, ischemic cardiomyopathy, and fibrogenic response to tissue injury (Lino Cardenas et al., 2013; Liu B. et al., 2016; Liu Y. et al., 2016; Zhang et al., 2016; Zuo et al., 2016; Zhou et al., 2017; Tian et al., 2018; Hromadnikova et al., 2019). More importantly, several studies observed that when the myocardium was in a hypoxic condition, the expression level of miR-199a-5p decreased rapidly, thus protecting the myocardium by adjusting its oxygen metabolism and angiogenesis *via* promoting the expression of target genes (Rane et al., 2009, 2010; Greco et al., 2012; Zhou et al., 2017). Recently, our study in mice exposed to chronic hypoxia demonstrated that inhibiting the expression of miR-199a-5p could subsequently alleviate endoplasmic reticulum stress (ERS) of the myocardium and avoid cellular apoptosis *via* upregulating the expression of its target genes, such as activating transcription factor 6 (ATF6) and 78 kDa glucose-regulated protein (GRP78) (Zhou et al., 2019). However, at high altitudes, whether miR-199a-5p might be involved in hypoxia-induced cardiovascular dysfunction and subsequent exercise capacity decline has been barely explored.

Therefore, in the present study, we examined the expression level of miR-199a-5p in plasma of 175 Han Chinese who had immigrated to high altitude regions 2 years previously. Moreover, we compared the expression level of miR-199a-5p between participants with good and poor exercise capacity at high altitude. Furthermore, we analyzed the association between miR-199a-5p, indices of cardiovascular function, and parameters of exercise capacity. Based on these observations and analyses, we speculate that the level of circulating miR-199a-5p can serve as an important indicator for cardiovascular fitness and exercise capacity during chronic high altitude exposure.

## Materials and Methods

### Participants

In total, 175 young healthy male lowlanders who had immigrated from sea level to high altitude regions (Shigatse, Tibet Autonomous Region, China, elevation: 3,800 m) 2 years previously were enrolled. They were all emergency rescue personnel with relatively good physical fitness due to regular training. All the participants were ethnic Chinese Han and aged between 18 and 27 years old. The protocol was approved by the Ethics Committee of Army Medical University and performed in accordance with the requirements of the Declaration of Helsinki. All participants provided written informed consent before they were enrolled.

### Study Procedures

**FIGURE 1:**
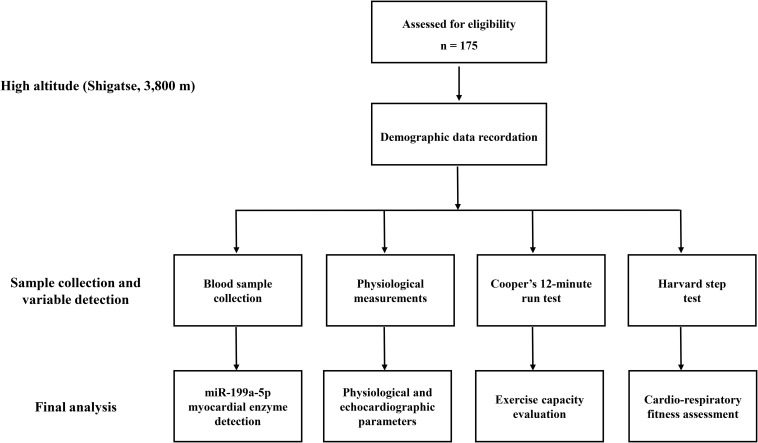
Trial flow diagram. VO_2_max, maximal oxygen uptake.

All the procedures were processed at 3,800 m with the help of medical staff at the local hospital. A day before the evaluation of aerobic exercise capacity (9:00 a.m.), demographic data and blood samples were collected. Then, the echocardiographic examination and physiological measurement were performed for all the participants. To evaluate the aerobic exercise capacity (9:00 a.m.), all participants carried out the exercise capacity test (Cooper’s 12-min run test). Then, after a six-hour rest, they next took a Harvard step test to assess cardiorespiratory fitness. During the investigation, medical monitoring and emergency first aid treatment were all available. The trail flow diagram of this study is shown in Figure 1.

### Clinical Data Collection and Physiological Measurement

A self-report questionnaire collected demographic data. The basic physiological parameters including pulse oxygen saturation (SpO_2_), heart rate (HR), diastolic blood pressure (DBP), and systolic blood pressure (SBP), were measured by physicians using a TuffSat Handheld Pulse Oximeter (GE Healthcare, Chicago, IL, United States) and sphygmomanometer (HEM-6200, OMRON, China). Then, after taking a 30-min rest, the echocardiographic indexes of the participants were recorded by sonographers using the color Doppler ultrasound system equipped with a 2.5 MHz adult transducer (Philip-CX50, United States), including cardiac output (CO), left ventricular ejection fraction (LVEF%), mitral E A peak flow velocity ratio (ME/A), the Tei index for left ventricular function (LV-Tei), mean pulmonary artery pressure (mPAP), and the Tei index of the right ventricle (RV-Tei). Sonographers of the local hospital performed all the measurements and calculations of the echocardiographic indexes.

### Plasma/Serum Collection and Storage

At 9:00 a.m., fasting venous blood samples were collected from participants with EDTA tubes and pro-coagulation tubes, respectively. Then, plasma and serum were separated by centrifugation at room temperature (speed: 3000 *g*, duration: 5 min). Finally, all the plasma and serum were transferred into RNase-free tubes and stored at −80°C.

### Cooper’s 12-Minute Run Test and the Definition of Exercise Capacity Grade

Participants underwent a 12-min run test on a flat and straight road. They were highly encouraged to run as fast as possible. Then, the finishing point of each participant was marked, and the total running distance in 12 min was calculated. To estimate the value of maximal oxygen uptake (VO_2_max), we employed Cooper’s equation [VO_2_max (mL/kg min) = 22.351 min × 12 min running distances (km) – 11.288] (Cooper, 1968). For the definition of exercise capacity grade, we followed the “*National Military Criteria of Evaluation of Physical Fitness for Soldiers at High Altitude*” (GJB 2559-1996 of China) (Gao et al., 2005) because the subjects were all professional emergency rescue team members with regular high-intensity exercise and physical fitness similar to that of soldiers. With a VO_2_max value of no less than 43 ml/kg min at 3,800 m, the participants were considered to possess good exercise capacity (GEC). On the contrary, if the VO_2_max value was under 43 ml/kg min, the participant was considered to have relatively poor exercise capacity (PEC).

### Harvard Step Test

The Harvard step test was used to assess cardiorespiratory fitness at a fixed load (Hanifah et al., 2014). Participants were asked to perform up-and-down exercises on a 40 cm high step at a frequency of 30 steps/min (the process of the left foot stepping up, the right foot stepping up, the left foot stepping down, and the right foot stepping down was counted as one step) for a continuous 5 min. The HR before stepping (Pre), immediately after stepping (Post), and during 5-min recovery were recorded by a chest-type HR monitor (POLAR RS800X, Wiggle, Finland). The Physical Fitness Index (PFI) was calculated by the HR recorded at 1.5 min (Post 1.5 min), 2.5 min (Post 2.5 min), and 3.5 min (Post 3.5 min) after stepping. The formula was as follows: PFI = duration time of the exercise (s) × 100/(HR Post 1.5 min + HR Post 2.5 min + HR Post 3.5 min). In addition, SpO_2_ before stepping, at the end of stepping, and during the 5-min recovery were detected by the TuffSat Handheld Pulse Oximeter (GE Healthcare, Chicago, IL, United States).

### Plasma miRNA Extraction and qRT-PCR Assay

miRNA was purified from the 200 μL plasma samples. At first, 3.5 μL of work solution of the miRNeasy Serum/Plasma Spike-In Control (*C. elegans* miR-39 miRNA mimic) (Cat.#: 219610, Qiagen, Valencia, CA, United States) was added as an endogenous control. Then, the total miRNA was extracted with the miRNeasy Serum/Plasma Kit (Cat.#: 217184, Qiagen, Valencia, CA, United States) according to the manufacturer’s instructions. Next, 6 μL of total miRNA was subjected to further reverse transcription and quantitative real-time PCR (qRT-PCR) reaction by commercial kits (Ribobio, Guangzhou, China) and the CFX Connect Real-Time PCR Detection System (Bio-Rad, United States). The calculation of the relative expression of miR-199a-5p was performed by the 2^–Δ*CT*^ method.

### Detection of Serum Myocardial Enzymes

The concentration of serum cardiac-specific troponin I (cTnI) was measured by the Cardiac Troponin Assay Kit (Cat.#: H149-2, Nanjing Jiancheng, Nanjing, China) based on the instructions of the manufacturer. To analyze the concentration of the serum MB isoenzymes of creatine kinase, the Human CK-MB ELISA Kit (Cat.#: ELH-CKMB, RayBiotech Life, Georgia, United States) was used following the manufacturer’s protocols.

### Biological Function Analysis of miR-199a-5p

MicroT-CDS v5.0 and TarBase v7.0 were employed to predict the target genes of miR-199a-5p (Paraskevopoulou et al., 2013; Vlachos et al., 2015a; Alhasan et al., 2016). Then, the above genes were enriched in gene ontology (GO) by DIANA-miRPath v3.0 (Vlachos et al., 2015b) using the following parameters: GO method (GO), subcategories (biological processes), species (human), and *p*-value threshold (0.05).

### Detection of Markers Associated With Oxidative Stress

The plasma concentration of markers associated with oxidative stress [malondialdehyde (MDA), superoxide dismutase (SOD), and heme oxygenase-1 (HO-1)] were determined with the Lipid Peroxidation MDA Assay Kit (Cat.#: S0131, Beyotime, Shanghai, China), Total Superoxide Dismutase Assay Kit (Cat.#: S0101, Beyotime, Shanghai, China), and Human HO-1 ELISA Kit (Cat.#: ml023053-C, Mlbio, Shanghai, China) according to the manufacturer’s instructions.

### Statistical Analysis

The Shapiro–Wilk test was utilized for checking the normality of variables. Then, the normally distributed variables were presented as mean ± standard deviation, and the median (interquartile range) was employed to exhibit non-normally distributed variables. In order to assess the difference of variables, if the variables were normally distributed between the GEC and PEC groups, the independent *t*-test was used, and if not, a Mann–Whitney *U* test was used. Cohen’s *d* value and *r* value [*r* = z/(sqrt N)] were determined as the effect size measures, respectively. Receiver operating characteristic (ROC) curves were calculated to evaluate the power of miR-199a-5p to distinguish PEC from the GEC groups. Spearman’s correlation was applied to analyze the relationship between variables, and the parameter of effect size was denoted by *r*. Statistical analyses were performed with IBM SPSS Statistics 19 (SPSS, Chicago, IL, United States). *P*-value ≤ 0.05 was considered statistically significant.

## Results

### Clinical Characteristics of Participants

For the distribution of participants with different grades of exercise capacity at 3,800 m, there were 113 participants in the GEC group, and the number of participants with PEC was 62. Compared with the GEC group, the PEC group performed worse in the 12-min running test (2.28 ± 0.17 km vs. 2.64 ± 0.16 km, *p* < 0.001, Cohen’s *d* = 2.181), and had a lower VO_2_max [39.62 ± 3.70 mL/(kg min) vs. 47.61 ± 3.53 mL/(kg min), *p* < 0.001, Cohen’s d = 2.210, Table 1].

**TABLE 1:** Clinical characteristics of participants (*n* = 175).

	GEC (113)	PEC (62)	Cohen’s d	*p* value
**Demographic data**				
Age (year)	21.04 ± 2.33	21.21 ± 1.85	0.081	0.236
Height (m)	1.73 ± 0.05	1.72 ± 0.05	0.200	0.898
Weight (kg)	63.41 ± 6.38	63.42 ± 7.69	0.001	0.992
BMI (kg/m^2^)	21.37 ± 1.99	21.32 ± 2.46	0.022	0.889
**Physiological parameters**				
SBP (mmHg)	119.05 ± 9.92	121.00 ± 10.33	0.192	0.269
DBP (mmHg)	86.58 ± 9.06	86.21 ± 12.84	0.033	0.839
HR (beat/min)	87.82 ± 10.61	87.72 ± 15.40	0.008	0.523
SpO_2_ (%)	90.30 ± 2.16	90.61 ± 2.74	0.126	0.283
**Echocardiographic indexes**				
CO (L/min)	5.08 ± 0.78	5.10 ± 0.84	0.025	0.871
LVEF (%)	69.50 ± 3.37	69.13 ± 2.96	0.117	0.474
ME/A	1.70 ± 0.49	1.72 ± 0.45	0.043	0.966
LV-Tei	0.38 ± 0.02	0.38 ± 0.03	0.005	0.182
mPAP (mmHg)	18.17 ± 2.18	21.32 ± 2.39	1.377	<0.001^#^
RV-Tei	0.19 ± 0.03	0.24 ± 0.04	1.414	<0.001^#^
**Myocardial enzyme**				
cTnI (pg/ml)	174.97 ± 28.84	206.35 ± 36.40	0.956	<0.001^#^
CK-MB (ng/ml)	4.21 ± 1.67	6.02 ± 2.90	0.765	<0.001^#^
**Exercise capacity variables**				
12 min running distance (km)	2.64 ± 0.16	2.28 ± 0.17	2.181	<0.001^#^
VO_2_max [mL/(kg min)]	47.61 ± 3.53	39.62 ± 3.70	2.210	<0.001^#^

*BMI, body mass index; CK-MB, MB isoenzymes of creatine kinase; CO, cardiac output; cTnI, cardiac-specific troponin I; DBP, diastolic blood pressure; GEC, participants with good exercise capacity at high altitude; HR, heart rate; LVEF%, left ventricular ejection fraction; LV-Tei, Tei index for left ventricular function; ME/A, mitral E A peak flow velocity ratio; mPAP, mean pulmonary artery pressure; PEC, participants with poor exercise capacity at high altitude; RV-Tei, Tei index of the right ventricle; SBP, systolic blood pressure; SpO_2_, pulse oxygen saturation; VO_2_max, maximal oxygen uptake.*

*Cohen’s d is an index for effect size.*

*^#^*p* value is 0.001 or less.*

Regarding demographic and basic physiological variables, there was no significant difference between the two groups. For the echocardiographic indexes, the PEC group had a higher value of mPAP (21.32 ± 2.39 mmHg vs. 18.17 ± 2.18 mmHg, *p* < 0.001, Cohen’s *d* = 1.377), and RV-Tei (0.24 ± 0.04 vs. 0.19 ± 0.03, *p* < 0.001, Cohen’s *d* = 1.414) compared with the GEC group, but no difference was found in left ventricular systolic or diastolic function (including CO, LVEF%, ME/A, and LV-Tei). For the value of serum myocardial enzymes, the PEC group had higher values of cTnI (206.35 ± 36.40 pg/mL vs. 174.97 ± 28.84 pg/mL, *p* < 0.001, Cohen’s *d* = 0.956), and CK-MB (6.02 ± 2.90 ng/mL vs. 4.21 ± 1.67 ng/mL, *p* < 0.001, Cohen’s *d* = 0.765), compared with the GEC group.

### Higher miR-199a-5p Level Accompanying Impaired Exercise Capacity at High Altitude

**FIGURE 2:**
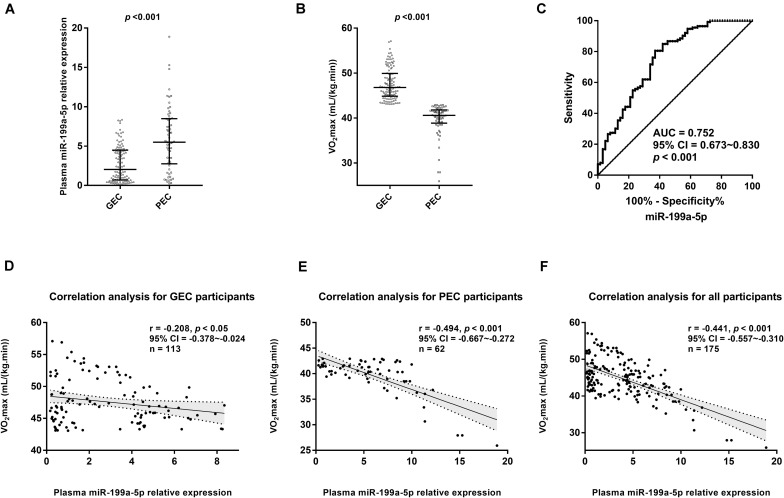
Circulating miR-199a-5p expressions were different between good exercise capacity (GEC) and poor exercise capacity (PEC) groups at high altitude. **(A)** Comparisons of plasma miR-199a-5p level in GEC and PEC groups. Effect size (r) for miR-199a-5p between two groupsis 0.416. **(B)** Comparisons of VO_2max_ values in GEC and PEC groups. Effect size (r) for VO_2max_ between GEC and PEC groupsis 0.826; **(C)** ROC curve analysis for plasma miR-199a-5p to discriminate PEC from GEC participants. **(D)** Correlation analysis forplasma miR-199a-5p level and *VO2max* value in GEC participants (*n* = 113). **(E)** Correlation analysis forplasma miR-199a-5p and *VO2max* in PEC participants (*n* = 62). **(F)** Correlation analysis forplasma miR-199a-5p and *VO2max* in all participants (*n* = 175). AUC: area under curve; CI: confidence interval; GEC: participants with good exercise capacity at high altitude; PEC: participants with poor exercise capacity at high altitude; VO_2max_: maximal oxygen uptake.

The qRT-PCR assay from 175 participants showed that compared with that in the GEC group, the plasma level of miR-199a-5p significantly increased in the PEC group, while VO_2_max obviously decreased (*p* < 0.001, Figures 2A,B). To reveal the relationship between the variables of the two groups, we utilized ROC curve and Spearman’s correlation analysis. The ROC curve demonstrated that miR-199a-5p was able to distinguish the PEC group from the GEC group (AUC = 0.752, *p* < 0.001, Figure 2C). Moreover, there was a significantly negative correlation between miR-199a-5p and VO_2_max both in the GEC (*r* = −0.208, *p* < 0.05, Figure 2D) and PEC groups (*r* = −0.494, *p* < 0.05, Figure 2E), as well as in all participants (*r* = −0.441, *p* < 0.001, Figure 2F).

### Higher miR-199a-5p Level Indicates Reduced Cardiorespiratory Reserve Function During Chronic Exposure to High Altitude

**FIGURE 3:**
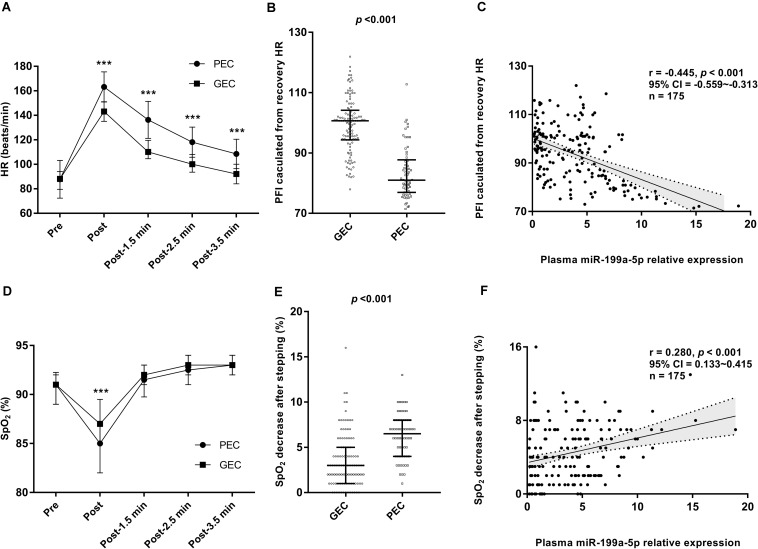
Relationship between miR-199a-5p expressions and cardiorespiratory reserve function at high altitude. **(A)** Comparisons of HR recovery after stepping test in GEC and PEC groups. Effect size (r) for HR-Post between GEC and PEC is 0.627, for HR-Post-1.5 min is 0.642, for HR-Post-2.5 min is 0.597, for HR-Post-3.5 min is 0.556. **(B)** Comparisons of PFI value in GEC and PEC groups. Effect size (r) between two groups is 0.649. **(C)** Correlation analysis forplasma miR-199a-5p expression level and PFI value in all participants (*n* = 175). **(D)** Comparisons of SpO_2_ recovery after stepping test in GEC and PEC groups. Effect size (r) between two groups is 0.330. **(E)** Comparisons of SpO_2_ decrease value after stepping test in GEC and PEC groups. Effect size (r) between two groups is 0.470. **(F)** Correlation analysis forplasma miR-199a-5p level and SpO_2_ decrease value after stepping test in all participants (*n* = 175). GEC: participants with good exercise capacity at high altitude; PEC: participants with poor exercise capacity at high altitude; PFI: physical fitness index; Pre: before stepping; Post 1.5 min: 1.5 minutes after the stepping; Post 2.5 min: 2.5 minutes after the stepping; Post 3.5 min: 3.5 minutes after the stepping. ***p value is 0.001 or less.

To analyze the relationship between the cardiorespiratory reserve function and the circulating miR-199a-5p level at 3,800 m, the Harvard step test was performed to compare the compensatory increase and the recovery of HR as well as the change of SpO_2_ during the stepping test. As shown in Figures 3A,B, the increase in HR under physical load was more obvious, and the recovery of HR was also slower in the PEC group, leading to a significant decline in the physical fitness index (PFI) which reflected relatively poor cardiorespiratory reserve. Moreover, Spearman’s correlation analysis confirmed a significantly negative association between miR-199a-5p and PFI (*r* = −0.445, *p* < 0.001, Figure 3C). The SpO_2_ decreased more obviously for the PEC group than that of the GEC group at the end of the 5 min stepping test (Figures 3D,E). Further relevance analysis among the 175 participants showed a positive correlation between the miR-199a-5p level and the reduced SpO_2_ following the stepping test (*r* = 0.280, *p* < 0.001, and Figure 3F). Together, these results exhibited that when participants’ miR-199a-5p expression level increased, the cardiorespiratory reserve function was more inadequate during chronic exposure to high altitude.

### Higher miR-199a-5p Level Correlated With Impaired Right Ventricle Function and Myocardial Injury During Chronic Exposure to High Altitude

**FIGURE 4:**
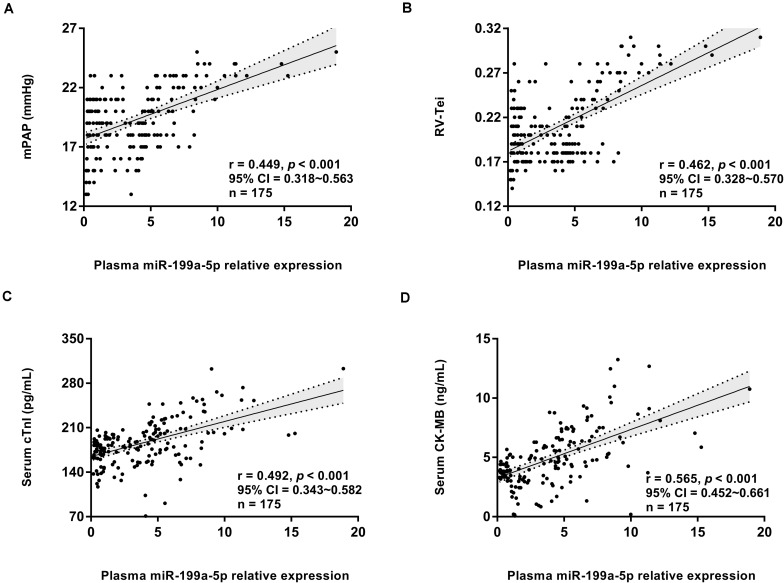
Circulating miR-199a-5p expression correlated with right ventricle function and myocardial injury at high altitude. **(A)** Correlation analysis forplasma miR-199a-5p level and mPAP value in all participants (*n* = 175). **(B)** Correlation analysis forplasma miR-199a-5p level and RV-Tei value in all participants (*n* = 175). **(C)** Correlation analysis forplasma miR-199a-5p level and serum cTnI concentration in all participants (*n* = 175). **(D)** Correlation analysis forplasma miR-199a-5p level and serum CK-MB concentration in all participants (*n* = 175). CK-MB: MB isoenzymes of creatine kinase; cTnI: cardiac specific troponin I; mPAP: mean pulmonary artery pressure; RV-Tei: Tei index of right ventricle.

A correlation analysis further revealed that miR-199a-5p expression level at high altitude was significantly positively correlated with the right ventricle function indexes, including mPAP and RV-Tei (Figures 4A,B), but had no correlation with the left ventricular indexes (including CO, LVEF%, ME/A, and LV-Tei). Meanwhile, the miR-199a-5p level exhibited a significantly positive association with the concentration of myocardial injury marker, including serum cTnI and CK-MB (Figures 4C,D). These data suggest that the increased myocardial damage and impaired right ventricle function caused by the disorder of miR-199a-5p expression might be a part of the mechanisms leading to decreased exercise capacity at high altitude.

### MiR-199a-5p Participated in the Processes of Cellular Nitric Oxide Metabolism and Stress Response

**FIGURE 5:**
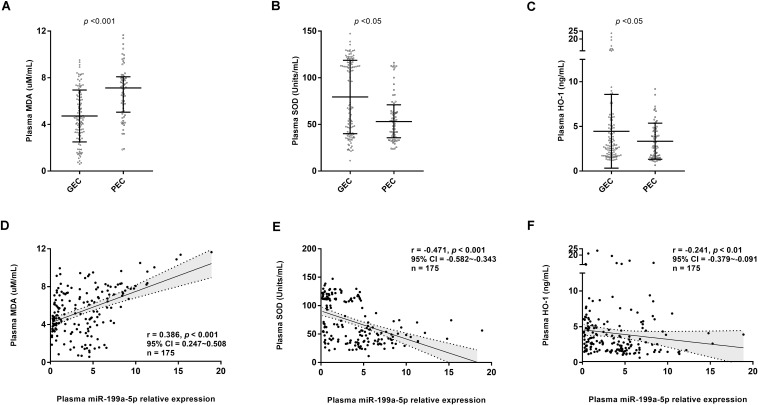
Circulating miR-199a-5p expression correlated withoxidative stress and antioxidant defense markers. **(A)** Comparisons of plasma MDA concentration in GEC and PEC groups. Effect size (Cohen’s d) between two groups is 0.937. **(B)** Comparisons of plasma SOD concentration in GEC and PEC groups. Effect size (r) between two groups is 0.253. **(C)** Comparisons of plasma HO-1 concentration in GEC and PEC groups. Effect size (Cohen’s d) between two groups is 0.366. **(D)** Correlation analysis forplasma miR-199a-5p level and MDA concentration in all participants (*n* = 175). **(E)** Correlation analysis forplasma miR-199a-5p level and SOD concentration in all participants (*n* = 175). **(F)** Correlation analysis forplasma miR-199a-5p level and HO-1 concentration in all participants (*n* = 175). HO-1: heme oxygenase-1; MDA: malondialdehyde; SOD: superoxide dismutase.

Gene ontology enrichment analysis showed that miR-199a-5p could regulate biological processes, such as the cellular nitrogen compound metabolic process, neurotrophin TRK receptor signaling pathway, stress response, and unfolded protein response (Table 2). Moreover, compared with the PEC group, the GEC group had a significantly lower expression level of oxidative stress marker (MDA) and a higher concentration of antioxidant stress markers (SOD and HO-1) (all, *p* < 0.05, Figures 5A–C). Furthermore, Spearman’s correlation analyses indicated that the level of miR-199a-5p exhibited a significantly positive relationship with MDA concentration and a negative association with SOD and HO-1 (all, *p* < 0.01, Figures 5D–F).

**TABLE 2:** GO enrichment for the target genes of miR-199a-5p.

GO ID	GO category	*p* value	No. of genes
GO:0034641	Cellular nitrogen compound metabolic process	1.26E–30	230
GO:0006950	Response to stress	2.28E–02	79
GO:0006987	Activation of signaling protein activity involved in unfolded protein response	4.70E–03	7
GO:0048011	Neurotrophin TRK receptor signaling pathway	4.84E–02	11
GO:0009058	Biosynthetic process	1.12E–20	189
GO:0006464	Cellular protein modification process	7.41E–08	99
GO:0010467	Gene expression	1.62E–10	39
GO:0043687	Post-translational protein modification	4.90E–05	14

*GO, gene ontology; NO., number.*

*The GO biological processes related to pulmonary arterial pressure and myocardial injury are listed in this table.*

## Discussion

The main finding of our study was that the level of plasma miR-199a-5p was closely associated with aerobic exercise capacity during chronic exposure to high altitude. Moreover, miR-199a-5p served as a novel biomarker for distinguishing individuals with PEC from those with GEC at high altitude. Furthermore, impaired right ventricle function (elevated mPAP and RV-Tei) and serious myocardial injury (higher cTnI and CK-MB) related to higher miR-199a-5p might have contributed to the decline of exercise capacity at high altitude.

To the best of our knowledge, this study described for the first time a link between circulating miR-199a-5p level and the aerobic exercise capacity of individuals during chronic exposure to high altitude. Moreover, our results also demonstrated that circulating miR-199a-5p was a novel biomarker for identifying individuals with PEC from those with GEC. It is of great significance to objectively evaluate aerobic exercise capacity while people are engaged in mountaineering, sports competition, commercial activity, and military operations at high altitudes, in order to decrease accidents and improve their work performance. Because it is difficult and dangerous to measure maximum exercise capacity by various exercise tests in a high altitude hypoxic condition, the index from body fluid reflecting cardiopulmonary endurance can be used as an alternative method with acceptable accuracy. Circulating miRNAs are considered specific, stable, and easily detectable biomarkers and have been applied successfully in diagnosing various diseases (Fung et al., 2019). In the current study, circulating miR-199a-5p displayed the potential to identify individuals with PEC from those with GEC at high altitude. As a novel and easily detectable molecule, miR-199a-5p is expected to become one of the reference indexes to evaluate and predict physical endurance at high altitudes in the future, contributing to accident prevention and work performance improvement in high altitude activity.

Enhancing cardiovascular function is an important way to improve exercise capacity at high altitudes (Swenson et al., 2014). miR-199a-5p is abundantly expressed in the cardiovascular system and acts as an important regulator for cardiovascular function (Haghikia et al., 2011). In this regard, we assessed the association between miR-199a-5p expression and the indices of cardiovascular function. The results demonstrated that higher miR-199a-5p expression was strongly associated with worse cardiovascular function (higher mPAP, lower right ventricular function, and more severe myocardial injury). Previous studies have indicated that higher pulmonary artery pressure and impaired right ventricular function limited exercise capacity for lowlanders during chronic exposure to high altitude (Naeije et al., 2010; Yang et al., 2015). Natives of Tibet, who are well-adapted to chronic hypoxia due to natural genetic evolution, have lower pulmonary artery pressure, better right ventricular function, and greater exercise capacity at high altitudes (Faoro et al., 2014). Recently, numerous studies have demonstrated that inhibiting miR-199a-5p expression *via* agents could alleviate pulmonary hypertension and myocardial injury, thus improving cardiovascular function (Rane et al., 2009; Liu B. et al., 2016; Liu Y. et al., 2016; Zhou et al., 2017, 2019). Recently, it was also reported that miR-199a was higher in patients with both ischemic and non-ischemic heart failure (De Rosa et al., 2018). Taken together, in the process of adapting to chronic hypoxia, lower mir-199a-5p should be beneficial to the heart, which happens in most people who live at a plateau. On the other hand, the level of mir-199a-5p is too high in a small number of people, which aggravates cardiovascular dysfunction, thus reducing exercise capacity at high altitude.

miRNAs exert their biological function by inhibiting the expression of target genes in a post-transcriptional manner. GO analysis of miR-199a-5p’s target genes has demonstrated that its target genes are enriched in biological processes, including the cellular nitrogen compound metabolic process, neurotrophin TRK receptor signaling pathway, response to stress, and unfolded protein response. Nitric oxide generated through the cellular nitrogen compound metabolic process is a major signaling and effector molecule mediating the body’s reaction to hypoxia (Beall et al., 2012). A higher level of NO promotes the adaption of native Tibetans and the acclimation of lowlanders to hypoxia *via* lowering pulmonary hypertension, preserving the cardiac function, and enhancing oxygen delivery (Erzurum et al., 2007; Janocha et al., 2011). Moreover, the Neurotrophin TRK receptor is upregulated by hypoxia and could induce NO generation in human pulmonary artery endothelial cells *via* binding to brain-derived neurotrophic factors (Meuchel et al., 2011; Helan et al., 2014). Recently, Joris V and Dessy C indicated that the inhibition of miR-199a-5p can increase NO bioavailability by promoting the activity of endothelial NO synthase both *in vivo* and *in vitro*, thereby modulating vascular contractile tone (Joris et al., 2018). Therefore, the decreased production of NO *via* higher miR-199a-5p might contribute to the higher pulmonary arterial pressure, which limits exercise capacity at high altitude.

The maintenance of normal cardiac function is highly dependent on oxygen availability. Under the hypoxic condition, excessive generation of reactive oxygen species caused oxidative stress injuries and sustained endoplasmic reticulum stress, thus leading to myocardial apoptosis and cardiac dysfunction (Zhou et al., 2017). During the process of hypoxia-induced myocardial injury, several endogenous protective mechanisms are activated to prevent cardiomyocytes from apoptosis, including upregulating antioxidant stress genes and promoting unfolded protein response. A previous study has confirmed that Sirtuin 1 (SIRT1) is a target gene for miR-199a-5p, and inhibiting miR-199a-5p could promote the expression of SIRT1 and decrease hypoxia-induced cardiomyocytes apoptosis (Rane et al., 2009). Moreover, SIRT1, the master regulator in response to oxidative stress, could activate the expression of several antioxidative genes to alleviate oxidative stress and rescue cellular apoptosis (Sosnowska et al., 2017). Consistently, our results also proposed that there was a strong negative association between miR-199a-5p and antioxidants (SOD and HO-1). Furthermore, unfolded protein response is another important protective process against hypoxia-induced stress, which could prevent cells from apoptosis *via* correcting and removing misfolded proteins (Wang et al., 2014). Importantly, two of our recent research papers confirmed that inhibition of miR-199a-5p could enhance the UPR process by promoting the expression of GRP78 and ATF6 both *in vivo* and *in vitro*, thereby avoiding chronic hypoxia-induced cardiomyocytes apoptosis and the subsequent impairment of cardiac function (Zhou et al., 2017, 2019). Taken all together, increased miR-199a-5p might contribute to hypoxia-induced pulmonary hypertension and myocardial injuries, thereby aggravating cardiovascular dysfunction and impaired exercise capacity at high altitude.

Most studies supported the opinion that a high level of miR-199a-5p was harmful to cardiovascular health, which was consistent with our data. But a few studies also reported opposite results. For example, the exogenous LIF and subsequent increased miR-199a might be beneficial to left ventricular function (Zgheib et al., 2012). These data seemed contradictory but logical because there were differences in the pathophysiological process between the left and right heart ventricles in disease, and the roles of miR-199a-5p during normoxia and hypoxia (or ischemia) might also be diverse. One piece of evidence was that the expression between ischemic and non-ischemic heart failure patients was distinguished (De Rosa et al., 2018). Therefore, the clinical significance of miR-199a-5p is complex, and should be considered according to the pathophysiological characteristics of the specific disease.

It should be noted that the correlation observed in this study only came from high altitude male migrants. Studies have shown that although the endurance of both men and women during high altitude hypoxia will be significantly reduced, there were some differences in the underlying mechanism. In terms of cardiopulmonary function, it was found that the exercise-induced arterial hypoxemia at high altitude was more closely related to the heart rate in men than that in women, while ventilation and energy expenditure contributed more in women (Horiuchi et al., 2019). In the aspect of oxygen transport and utilization, it was reported that although the hemoglobin content of women was lower than that of men, the affinity between oxygen and hemoglobin was weaker, which was conducive to the release of oxygen to tissue (Balcerek et al., 2020). So far, the existing data showed that there was no significant difference in circulating mir-199a-5p levels between men and women regardless of physiological and disease conditions (Sanfiorenzo et al., 2013; Cammarata et al., 2018). However, due to gender differences in the detailed mechanism underlying declined aerobic exercise tolerance at altitude, there might be differences between men and women in how and to what extent mir-199a-5p affected exercise ability, as well as the significance of mir-199a-5p as a biomarker. To clarify these issues, female subjects need to be included in future studies.

### Limitations

There were two limitations to our study. Firstly, the sample size was relatively small, owing to the difficulty in recruiting subjects. Secondly, all participants were young, male, and Chinese Han because they made up the leading group of lowlanders who immigrated to high altitude regions. Thirdly, this study lacked a baseline value of miR-199a-5p before going to high altitude and the individual change of miR-199a-5p after high altitude hypoxia, so it could not be excluded that other confounding factors influenced the background level of miR-199a-5p. Thus, there is a need for testing a larger group of individuals with diverse gender, age, and ethnicity. In particular, it should detect more complete data, especially miR-199a-5p before and after entering high altitudes.

## Conclusion

In this study, we reported for the first time that circulating miR-199a-5p was associated with exercise capacity at high altitudes and served as a novel potential biomarker for indicating poor exercise endurance. Moreover, increased miR-199a-5p might be involved in hypoxia-induced cardiovascular dysfunction, thus contributing to the reduction in exercise capacity at high altitude.

## Data Availability Statement

The raw data supporting the conclusions of this article will be made available by the authors, without undue reservation.

## Ethics Statement

The studies involving human participants were reviewed and approved by the Army Medical University (Third Military Medical University) Ethics Committee, China. The patients/participants provided their written informed consent to participate in this study.

## Author Contributions

PL and YG conceived and designed the study. BX oversaw laboratory analyses. PL provided the overall supervision of the study. HH, ZZ, and PH completed the statistical analysis or contributed to the laboratory experiments. SX and XG contributed to sample and data collection. HH drafted the report. PL and YG were the guarantors. All authors contributed to the interpretation of the results, critical revision of the manuscript, and approved the final manuscript.

## Conflict of Interest

The authors declare that the research was conducted in the absence of any commercial or financial relationships that could be construed as a potential conflict of interest.
